# An Unusual Case of a Large Extraovarian Endometrioma in an Adolescent

**DOI:** 10.1155/2022/1675353

**Published:** 2022-07-08

**Authors:** Wei-Guo Nicholas Loh, Nira Borok, Lyndal Anderson, Tanushree Rao

**Affiliations:** ^1^Obstetrics and Gynaecology Department, Liverpool Hospital, Locked Bag 7103 Liverpool NSW 2170 Sydney, Australia; ^2^University of New South Wales, South Western Sydney Clinical School, Liverpool Hospital, Level 2, Clinical Building, Locked Bag 7103 Liverpool NSW 2170, Sydney, Australia; ^3^Radiology Department, Liverpool Hospital, Locked Bag 7103 Liverpool NSW 2170, Sydney, Australia; ^4^Anatomical Pathology Department, Royal Prince Alfred Hospital, PO Box M30, Missenden Road NSW 2050, Sydney, Australia

## Abstract

Endometriosis in adolescents is often underrecognized and is a contributing factor to significant delays in its diagnosis and management. Extraovarian endometrioma is uncommon, especially in the adolescent age range. We report on a rare case of a large extraovarian endometrioma involving the terminal ileum in an adolescent who presented with abdominal pain and a pelvic mass and its subsequent surgical management. It emphasizes the importance of having a broad differential diagnosis and considers endometriosis in any adolescent with pelvic pain and mass, especially in an atypical context. Increased awareness, education, and research on endometriosis in this young population are essential in order to overcome existing challenges in the early diagnosis and optimal management of this chronic gynaecological condition and avoid morbidity associated with advanced disease.

## 1. Introduction

Endometriosis is a common gynaecological disorder characterised by the presence of ectopic endometrial tissue outside of its usual location within the uterus [1]. These tissues are most commonly found on dependent surfaces within the pelvis, in particular on the ovaries, which often result in the formation of endometriotic cysts (also known as endometriomas). The rare involvement of endometriosis in distal extrapelvic sites has also been previously documented [2]. Endometriosis and endometriomas in adolescents are however less well characterised compared to those in adults and often underrecognized [1, 3, 4]. We report on a rare case of a large extraovarian endometrioma in an adolescent and its management. Written informed consent from the patient was obtained for the preparation and publication of this report.

## 2. Case Presentation

A 19-year-old nulliparous female was referred to the emergency department with a computed tomography scan showing a large right adnexal lesion 9.5 × 6.5 × 6 cm associated with moderate free fluid, adjacent fat stranding, and adjacent normal appendix. This was on the background of a one-day history of severe right iliac fossa colicky pain that was not associated with any other gastrointestinal, genitourinary, or infective symptoms and was not relieved by regular paracetamol and ibuprofen. She had no significant past medical history or family history of the disease and was a nonsmoker and nondrinker. She has an unremarkable regular 28-day menstrual cycle, with her last menstrual period 1 week prior. She was sexually active in a monogamous long-term relationship with her male partner of 17 months, with no history of sexually transmitted infection, and was on the combined oral contraceptive pill.

On examination, there was suprapubic tenderness without peritonism and right adnexal tenderness without cervical excitation. Investigations showed WCC 13 × 10^9^/L and CRP 5 mg/L, with normal renal and liver function, and her tumour markers (CA-125, 19.9, CEA, AFP) were normal. Her STI screen was negative. Further evaluation of the lesion with a transvaginal pelvic ultrasound showed a nonspecific heterogenous hyperechoic lesion with small cystic spaces in the right adnexa which appeared inseparable from the right ovary. Her pain settled following admission, and she was discharged with follow-up plans in the gynaecology clinic for further imaging and surgical planning.

**Figure 1:**
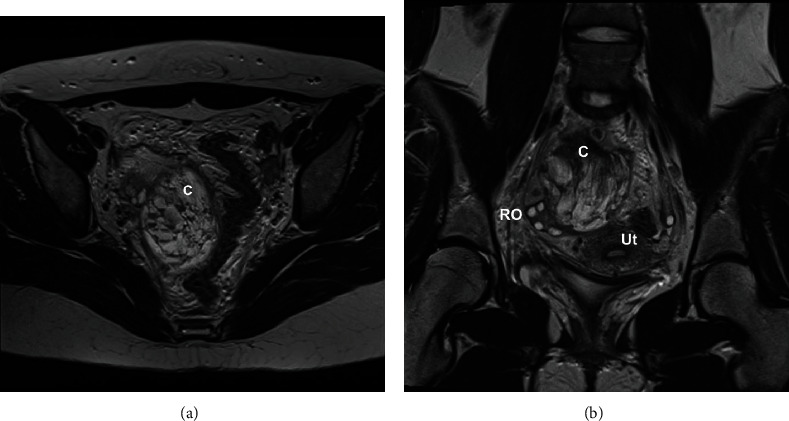
MRI of the pelvis. (a) (11) Axial T2-weighted image demonstrating the large complex cystic mass (C) located at the right hemipelvis. The lesion is multiloculated with internal haemorrhage seen as fluid-fluid levels and T2 shading (darker cyst content). (b) Coronal T2-weighted image with the normal right ovary (RO) is demonstrated lateral to the complex cystic mass (C). Large irregular low T2 signal intensity regions within the cystic lesion relate to blood products. Ut: uterus.

She was readmitted 4 days later with right iliac fossa pain, vomiting, and CRP elevated to 93 mg/L. CA-125 was mildly elevated at 57 kU/L. She underwent a pelvic MRI which showed an indeterminate large right adnexal lesion with large areas of low T1/T2 signal intensity, multiple small cysts, and no enhancement (Figure 1). Several of the cysts had fluid levels, and there was marginal T1 high signal at the inferior aspect thought to relate to blood products. The adjacent right ovary had a normal appearance. There were no features to suggest a high-grade neoplasm. The peritoneal fluid showed T1 hyperintensity consistent with hemoperitoneum. She was counselled and consented for a diagnostic laparoscopy +/- detorsion of the paraovarian lesion, with an aim for fertility sparing surgery.

**Figure 2:**
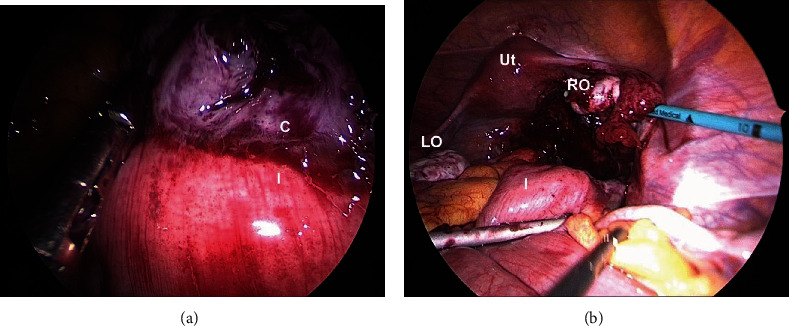
Intraoperative images obtained during laparoscopic cystectomy. (a) Cyst wall (C) firmly adherent to terminal ileum (I). (b) Normal uterus (Ut) and left and right ovaries (LO, RO) demonstrated postcystectomy.

Operative findings revealed a large cyst that was separate from the normal-looking right ovary and was firmly adherent to the pouch of Douglas, right pelvic sidewall, terminal ileum, and sigmoid colon (Figure 2). The pelvic organs were otherwise unremarkable, and there were no other signs of pelvic endometriosis or ovarian torsion. The chocolate fluid from inadvertent surgical rupture of the cyst suggested that the lesion was likely to be consistent with an endometrioma. A combination of blunt and sharp dissection was used to separate the pelvic cyst from its surrounding attachments. A residual portion of the thin 2 × 2 cm cyst wall was identified densely adhering to the terminal ileum, necessitating a colorectal consult. A decision was made to leave the cyst wall behind as the risk of significant bowel injury and complications outweighed the benefit of complete cystectomy.

**Figure 3:**
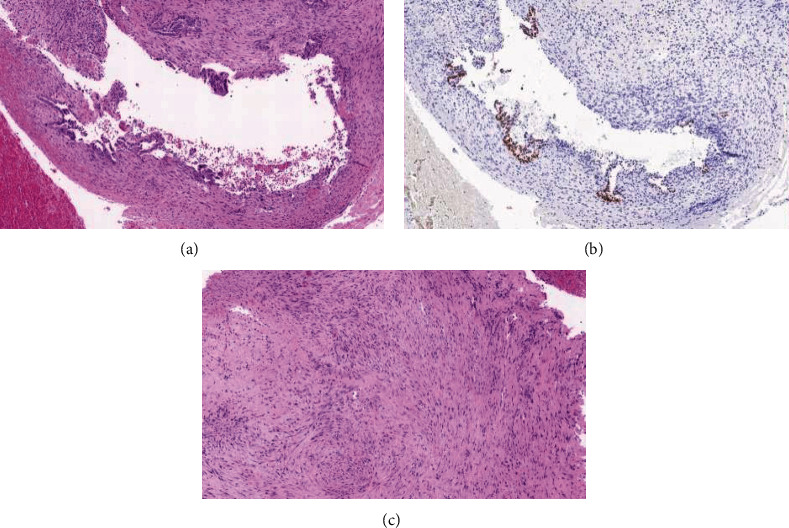
Histopathology. (a) Endometrioid epithelium showing reactive-type changes. (b) Estrogen receptor immunohistochemistry positivity in the endometrioid epithelium. (c) Stromal component showing fibrosis.

Her postoperative recovery was unremarkable, and she was discharged home the following day with outpatient gynaecology clinic follow-up. Histopathology confirmed that the lesion was a fibrotic endometrial cyst with mesothelial hyperplasia, without evidence of malignancy (Figure 3). Pelvic washings were negative for malignant cells. She was subsequently commenced on hormonal therapy for the management of endometriosis and has ongoing colorectal and gynaecology follow-up given the potential risk of malignant transformation of the remaining cyst wall on the terminal ileum.

## 3. Discussion

Endometriosis is a common condition estimated to affect up to 15% of all reproductive aged women. The age range is highly variable with isolated case reports of fetal endometriosis. Adolescents with endometriosis often report a delay in clinical diagnosis. The prevalence of endometriosis in adolescents was found to be as high as 62% of all those who undergo laparoscopy for pelvic pain [5]. Moreover, literature on the clinical characteristics of endometrioma specifically in adolescents has been fairly limited compared to adults, with few studies published to date [3, 4, 6]. Extraovarian endometriomas are also exceedingly uncommon [7, 8]. In this report, we describe the clinical presentation and management of the first ever case of an extraovarian endometrioma in an adolescent.

The aetiology of endometriosis has yet to be clearly elucidated. One of the most accepted theories in the pathogenesis of the disease involves the implantation of endometrial tissue on the dependent surfaces of the pelvic cavity via retrograde menstruation through patent fallopian tubes [1]. This theory is further supported by studies showing that Müllerian anomalies resulting in outflow tract obstruction were associated with the development of endometriosis at a young age [5]. Other theories on the pathogenesis of endometriosis include coelomic metaplasia, haematogenous dissemination, or lymphatic dissemination [1, 9] which may explain the presentation in unusual sites such as the lung and lymph nodes. The patient did not have any history supportive of underlying Mullerian anomalies. The location of this extraovarian endometrioma suggests either retrograde menstruation or coelomic metaplasia to be the most likely cause in this case.

The diagnosis of endometriosis in adolescents continues to present a significant challenge for various reasons such as (i) patient and physician knowledge gap and normalisation of symptoms, particularly when adolescents usually present atypically with noncyclical pain; (ii) the lack of research specific for adolescents; (iii) and a higher threshold for laparoscopic surgery for definitive diagnosis [10]. Recent studies suggested that advanced-stage endometriosis and endometriomas might be more common in adolescents than we previously understood, but the current literature in adolescents still pales in comparison to that of adults [5]. Ovarian endometriomas can have a detrimental effect on ovarian function and reserve with time via inflammation-mediated fibrosis of the ovarian cortex [1]. As such, the timely diagnosis of an ovarian endometrioma is of particular significance in an adolescent.

Surgery has traditionally been the main definitive treatment for endometrioma when surveillance is not appropriate [11]. Surgical treatment of early-stage endometrioma is suggested to provide symptom relief and increase quality of life and decrease the potential negative impact of the disease on future fertility, while surgical intervention is recommended for endometriomas of 6 cm or greater due to the associated risks of infection, rupture, and malignancy [1, 9]. Recent small cohort studies have however reported encouraging results of reduction in endometrioma size with oral Dienogest, with one study reporting a 40% and 79% reduction after 6 and 12 months of treatment, respectively [12–15]. Future research involving larger well-designed studies with a longer-term follow-up data in this area can provide the much-needed quality evidence to support the emerging role of Dienogest in the primary treatment of endometrioma. In our case, we proceeded with fertility-preserving surgery given clinical concerns with ovarian torsion. It was interesting to note that the lesion was separate from the presumed affected ovary and was most firmly adherent to the terminal ileum.

A large retrospective cohort study previously reported that the recurrence rate of ovarian endometrioma in adolescents after primary surgery increases over time, with a relatively low recurrence rate of 6.4% in the short term (24 months), but 30.9% at 96 months [6]. Notably, the incomplete removal of endometrioma tissue due to surgical techniques such as piecemeal removal or ablation of the cyst capsule is associated with increased rates of recurrence [16–18]. As such, the risk of recurrence in our case is considered high however, given that we were unable to completely excise the remainder of the cyst wall from the terminal ileum without significant risk of bowel complications. Ongoing follow-up has been arranged to monitor the patient's symptoms and recurrence of the disease, risk of bowel complication with residual disease on the terminal ileum, and the small risk of malignant transformation over time [1, 19]. The evidence behind postoperative hormonal suppression to prevent recurrence remains unclear [1, 5, 6]. The patient has continued on the combined oral contraceptive postoperatively.

In conclusion, this is a rare case of a large extraovarian endometrioma in an adolescent. It emphasizes on the importance of having a broad differential diagnosis and considers endometriosis in any adolescent with pelvic pain and mass, especially in an atypical context. Increased awareness, education, and research on endometriosis in this young population are essential in order to overcome existing challenges to allow for early diagnosis and treatment and avoid morbidity associated with advanced disease.

## Data Availability

The clinical images used to support the findings of this study are included in the article.
